# A Fast Estimation Method for Direction of Arrival Using Tripole Vector Antenna

**DOI:** 10.3390/s20175008

**Published:** 2020-09-03

**Authors:** Bodong Zhang, Xuan Zou, Tingyi Zhang, Yunong Tang, Hao Zeng

**Affiliations:** School of Communication Engineering, Chongqing University, Chongqing 400044, China; 20173633@cqu.edu.cn (B.Z.); 20173646@cqu.edu.cn (X.Z.); 20143980@cqu.edu.cn (T.Z.); 201912131119@cqu.edu.cn (Y.T.)

**Keywords:** DOA estimation, polarization, vector antenna

## Abstract

The tripole vector antenna comprises three orthogonal dipole antennas, so it could completely capture all the electric field of the incident electromagnetic (EM) wave. Then, the electric field information could be used to estimate the direction of arrival (DOA) of the EM wave if two conditions are satisfied. One is that there exists only one single EM wave in space. The other is that the EM wave is elliptically or circularly polarized. The new estimation method obtains two snapshot vectors through the output of a tripole antenna and computes their cross-product vector. Furthermore, the direction of the cross-product vector is used to estimate the DOA of the EM wave directly. We analyze the statistical characteristics of the DOA estimation error to prove that the new scheme is an asymptotic unbiased estimation. Furthermore, unlike the existing Multiple Signal Classification (MUSIC)-based algorithms, the proposed approach only need one tripole vector antenna instead of an antenna array. Meanwhile, the new method also outperforms existing MUSIC-based algorithms in the term of computational complexity. Finally, the performance and advantages of the proposed method are verified by numerical simulations.

## 1. Introduction

The incident angle of the electromagnetic (EM) wave relative to the receiver is called direction of arrival (DOA). In radar, communication, radio astronomy, telemetry, track and command, and other fields [1,2,3], users always need to estimate the DOA of EM waves. For example, the DOA information wave can be used in the beam control system of the receiver antenna, so that the main beam of the receiver antenna can track the incident signal all the time. Different DOA estimation methods are adopted in various application fields. Each method has its own advantages and keeps developing continuously.

Nowadays, the most traditional DOA estimation equipment is the interferometer, which estimates the DOA according to the phase difference between EM waves received by adjacent antenna elements. The phase difference is caused by the difference in distance between the radiation source and different antenna elements, and it has a certain geometric relationship with the incident angle. However, there are some shortcomings in DOA estimation of the interferometer. Firstly, the estimation accuracy is not high because the algorithm is vulnerable to noise. Obtaining the phase difference is a key step for interferometer. Common methods include zero-crossing detection, Fast Fourier Transform (FFT) [4], and Hilbert orthogonal transformation [5] and so on. The zero-crossing detection uses the approximation of the function near the zero point, i.e., sin*x* = *x*, to accurately determine the zero-crossing position of the sample sequence. These positions can help to calculate the path difference between the signals received by different antenna elements. Unfortunately, noise results in serious uncertainty when estimating the zero point. In addition to noise, the estimation accuracy is also related to the sample frequency of the signal, which requires high performance of the hardware. FFT is another way to estimate the phase difference between signals. The phase difference between the two signals can be easily obtained by FFT and cross-correlation operation on the sample sequence. Compared with zero-crossing detection, the FFT-based algorithm performs much better in anti-noise and anti-harmonic interference, so it could achieve higher accuracy in the phase difference estimation. The second shortcoming for the interferometer is that the estimation accuracy is closely related to the array spacing. The shortest interval in an interferometer array is usually less than half a wavelength; hence, the phase measured in this case is unique [6,7]. However, in broadband direction-finding systems over a range such as 0.1–3 GHz or 2–8 GHz, the physical dimensions of the antenna element are dictated by the lowest operating frequency, whereas the shortest interelement spacing is decided by the highest frequency. This poses the problem of not being able to employ a short spacing of less than half-wavelength [8,9]. However, if the baseline length exceeds the half-wavelength of the pulse signal, the periodic ambiguity will be introduced into the phase measurement and its ambiguity is 2π. The combination of long baseline and short baseline is an effective method to solve the phase ambiguity. Even so, the combination of multiple baselines greatly increases the complexity of the system and requires more accurate calibration of the system. Thirdly, the interferometer can only aim at a single signal in the main lobe, and cannot be used when multiple EM waves arrive at the antenna at the same time. In this case, it means insufficient resolution.

In order to promote the resolution and realize the DOA estimation of multiple incident signals, various methods based on antenna arrays have become the research hotspot in the last decades. So far, several classes of DOA estimation techniques for incoherently distributed sources have been developed, such as the subspace method and the parameter method. Multiple Signal Classification (MUSIC) and Estimating Signal Parameter via Rotational Invariance Techniques (ESPRIT) are the most important subspace methods [10,11,12]. Furthermore, the maximum likelihood (ML) algorithm [13,14] and Covariance Matching Estimator (COMET) algorithm [15,16] are typical parameter estimation approaches, which can obtain better estimation performance than subspace methods. However, their computing load is also heavier. Meanwhile, DOA estimation methods based on antenna arrays are facing two great challenges. First, the complexity of the algorithm is too high to implement from an engineering perspective. In these technologies, most methods are proposed for uniform linear arrays; therefore, the spectral peak search happens only on a two-dimensional plane, which makes it impossible to estimate the elevation angle and azimuth angle simultaneously. Later, researchers further developed these algorithms to make them suitable for planar arrays. In this way, the range of the spectral peak search is extended to three-dimensional space, which means that the DOA of the signal source can completely include both the elevation angle and azimuth angle. However, due to multi-dimensional space spectrum search, a large amount of computational burden has always been a problem that researchers need to solve [17]. In addition to the huge computation of the spectral peak search, this kind of DOA algorithm involves various complex operations such as matrix eigenvalue decomposition, which resulting in difficulties in the engineering implementation of programmable devices, such as FPGA. Another challenge is that these antenna arrays generally have a large number of elements and each element needs a corresponding radio frequency front-end channel, which makes the hardware cost very high.

Different from the popular algorithms mentioned above, we use a tripole vector antenna to make full use of the electric field of the EM wave and develop a fast DOA estimation algorithm for a single signal. The tripole vector antenna is also known as a polarization sensitive antenna, which is different from the traditional single polarization antenna because it can accurately sense the polarization characteristics of EM waves [18]. The tripole vector antenna is proposed by R.T Compton [19,20], and it is composed of three orthogonal dipole antennas. Document [21] proposed a DOA estimation method based on polarization-spatial steering vector. It uses a tripole antenna but needs to know the elliptical susceptibility of the polarized signal. In recent years, polarization-sensitive arrays have become the focus of academic research, where each element is one tripole vector antenna. Researchers try to achieve higher performance through polarization-sensitive arrays in the fields of spatial spectrum estimation and adaptive anti-jamming array. In fact, it is a signal processing method combining spatial and polarimetry resources, which improves the array freedom degree of the receiver. For example, the tripole antenna has been proposed as one possible choice of antenna construction to be used in the low-frequency array (LOFAR) project [22]. The primary field of application for the project is radio-based astrophysics.

Due to the structural characteristics of the tripole vector antenna, we only need to use one tripole vector antenna to complete the DOA estimation of the EM wave if there is only one signal in space. We map the sample data of the tripole vector antenna to the space rectangular coordinate system and calculate the DOA by vector product according to the geometric relationship. The new method has some obvious advantages over the other methods. First of all, the proposed DOA algorithm does not need to solve equations such as the interferometer or search the spectral peak such as array signal processing methods [23]; as a result, the signal processing process is much simpler. Secondly, the main lobe of the tripole vector antenna is wide enough so that any signal impinging from the main lobe area could be captured and no servo system is needed. Obviously, this reduces the cost of hardware greatly. Thirdly, the DOA estimation method based on the tripole vector antenna is suitable for any nonlinearly polarized EM wave without any polarization-matching problem between antennas and EM waves, because it captures all the polarization information of EM waves. This is also the advantage that other DOA estimation methods do not have. Similar to the interferometer, the new method also has the disadvantage of insufficient resolution, but it can be solved by combining with other subspace estimation algorithms.

The rest of this paper is as follows. Section 2 is a brief introduction of the received signal model. Section 3 is the overall description of the method. The method complexity and error analysis are discussed in Section 4. The experimental results of different methods are presented in Section 5. Section 6 concludes the paper.

## 2. Signal Model

**Figure 1:**
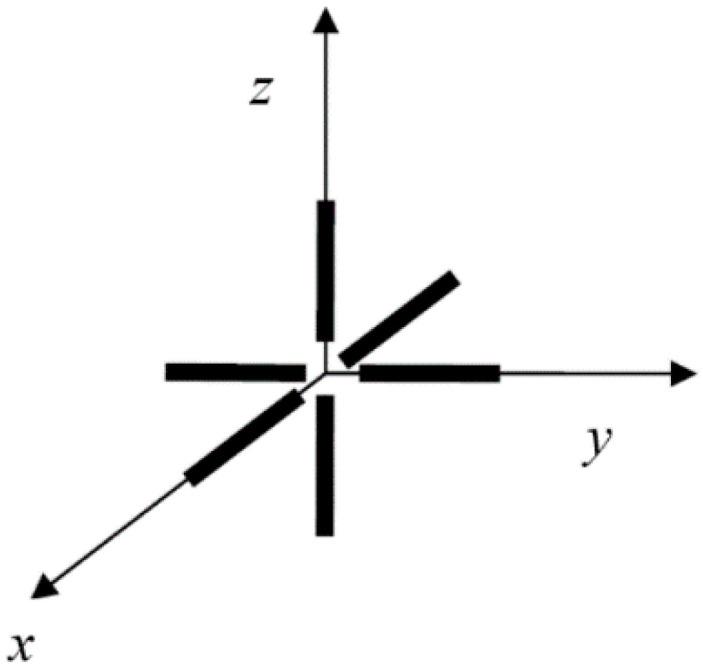
Structure of tripole vector antenna.

The tripole vector antenna is composed of three identical and mutually perpendicular linearly polarized dipole antennas centered at the same position [19,20,21], as shown in Figure 1. Each linearly polarized dipole antenna can only receive the electric field component in its polarization direction and has an output feed point. The polarization direction of the dipoles in a tripole are respectively parallel to the three coordinate axes of the space rectangular coordinate system, which ensures that no matter which direction the EM wave is incident from, and no matter what polarization the EM wave is, all the energy of the EM wave can be received by the tripole vector antenna. So, there will be no polarization-matching problem. In addition, since all three feed points are concentrated at the same location, phase shifts between units will not occur in a tripole vector antenna.

In the coordinate system shown in Figure 1, we suppose that the DOA of an elliptically polarized EM wave signal is incident from a certain direction. Once the signal reaches the antenna, its electric field will be simultaneously received by the three orthogonal dipoles of the antenna. We name the three poles of the antenna as *x*, *y*, *z*, hence the signal received by the antenna can be expressed as

(1) $$\begin{array}{ll} x\left(n\right) & =E_{x}\left(n\right)+n_{x}\left(n\right)\\ & =E_{xm}\cos\left(\omega nT_{s}+\varphi_{x}\right)+n_{x}(n) \end{array}$$

(2) $$\begin{array}{ll} y\left(n\right) & =E_{y}\left(n\right)+n_{y}(n)\\ & =E_{ym}\cos\left(\omega nT_{s}+\varphi_{y}\right)+n_{y}(n) \end{array}$$

(3) $$\begin{array}{ll} z\left(n\right) & =E_{z}\left(n\right)+n_{z}(n)\\ & =E_{zm}\cos\left(\omega nT_{s}+\varphi_{z}\right)+n_{z}(n) \end{array}$$

where *E_xm_*, *E_ym_*, and *E_zm_* and *ϕ_x_*, *ϕ_y_*, and *ϕ_z_* are the amplitudes and initial phases of the electric field components in the *x*, *y*, and *z* directions. *n_x_*(*n*), *n_y_*(*n*), and *n_z_*(*n*) are independent white Gaussian noise in the *x*, *y*, and *z* directions. *n* is the sample number, *T_s_* is the sample period, and *ω* is the angular frequency of the EM wave.

## 3. DOA Estimation Algorithm

### 3.1. Principle of Algorithm

For an elliptically polarized EM wave, according to the signal model, in the antenna coordinate system, the electric field of the received signal can be expressed as

(4) $$E\left(n\right)=x\left(n\right)e_{x}+y\left(n\right)e_{y}+z\left(n\right)e_{z}$$

where E(n) is the electric field of the received signal, and ***e****_x_*, ***e****_y_*, and ***e****_z_* are unit direction vectors in the *x*, *y*, and *z* directions respectively.

According to the definition of elliptically polarized electromagnetic waves, the electric field vector ***E***(*n*) is orthogonal to the propagation direction, and its trajectory is elliptical when viewed from the direction of propagation. Obviously, the output vector of the tripole vector antenna is equal to the electric field vector ***E***(*n*) at the origin of the coordinate. In addition, the plane of the trajectory must be perpendicular to the incident direction of the EM wave. According to the definition of the vector operation, the cross-product of two non-parallel vectors is perpendicular to the plane where they are located. Therefore, we can calculate the direction vector of the incident EM wave through these two snapshots. Of course, this also applies to circular polarization, because circular polarization is only a special case of elliptical polarization.

### 3.2. Caculation of Direction Vector

**Figure 2:**
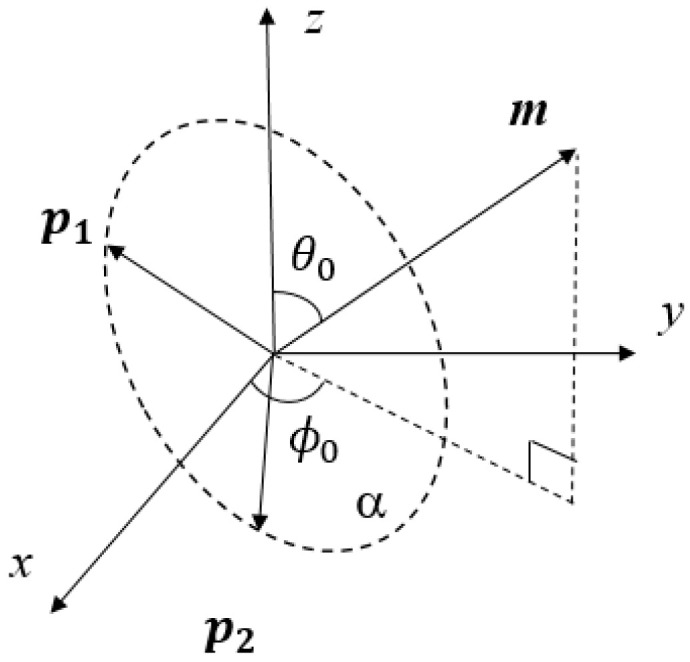
Normal vector diagram.

In the space rectangular coordinate system as shown in Figure 2, without considering noise, the electric field trajectory is an ellipse in the plane α. Two continuous sample snapshots of the tripole vector antenna are taken to describe two electric field vectors ***p***_1_ and ***p***_2_ at two different moments, which are

(5) $$p_{1}={[\begin{array}{ccc} E_{x}\left(2k\right) & E_{y}\left(2k\right) & E_{z}\left(2k\right) \end{array}]}^{T}$$

(6) $$p_{2}={\left[\begin{array}{ccc} E_{x}\left(2k+1\right) & E_{y}\left(2k+1\right) & E_{z}\left(2k+1\right) \end{array}\right]}^{T}$$

where symbol “*T*” means transposition. 2*k* and 2*k* + 1 are two continuous sample indexes. 

Apparently, as shown in the Figure 2, the angle between ***p***_1_ and ***p***_2_ is certainly less than π and greater than 0 because of the Nyquist sample theorem. Therefore, these two vectors are not on the same line. According to vector operation principle, the normal vector of the plane *α* can be represented by the cross product of ***p***_1_ and ***p***_2_, which is

(7) $$m\left(k\right)=p_{1}\times p_{2}={\left[\begin{array}{ccc} m_{x} & m_{y} & m_{z} \end{array}\right]}^{T}$$

where

(8) $$m_{x}=E_{y}\left(2k\right)E_{z}\left(2k+1\right)-E_{z}\left(2k\right)E_{y}\left(2k+1\right)$$

(9) $$m_{y}=E_{z}\left(2k\right)E_{x}\left(2k+1\right)-E_{x}\left(2k\right)E_{z}\left(2k+1\right)$$

(10) $$m_{z}=E_{x}\left(2k\right)E_{y}\left(2k+1\right)-E_{y}\left(2k\right)E_{x}\left(2k+1\right).$$

Obviously, the vector ***m*** is orthogonal to the plane α, since it is the cross-product of ***p***_1_ and ***p***_2_. Then, the direction of the vector ***m*** is parallel to the DOA of the EM wave, and that means we can estimate the DOA by computing the angle of vector ***m***. From the engineering point of view, we only consider incident EM waves from the upper hemisphere. Therefore, for incident EM waves from the lower hemisphere, all coordinates of vector ***m*** are inverted before the DOA estimation.

### 3.3. Noise-Free Angle Estimation Algorithm

For convenience, we assume temporarily that the received signal contains no noise. According to Figure 2, the elevation angle *θ*_0_ (*θ*_0_ ∈ [0, π/2]) is the angle between the vector ***m*** and the *z* axis. The azimuth angle *ϕ*_0_ is the angle between the projection of ***m*** in the *xy* plane and the *x* axis. If this projection is in quadrant 1 or 2, then *ϕ*_0_ ∈ [0, π], and if it is in quadrant 3 or 4, then *ϕ*_0_ ∈ [−π, 0]. From Figure 2, the elevation angle $\theta_{0}$ should satisfy.

(11) $$\cos\theta_{0}=\frac{m_{z}^{}}{\sqrt{m_{x}^{2}+m_{y}^{2}+m_{z}^{2}}}$$

The value of Equation (11) is always not negative because we only consider EM waves coming above the *xy* plane; thus, the estimated elevation angle *θ*_0_ can be calculated by the inverse cosine function, which is

(12) $${\hat{\theta }}_{0}=\arccos\left(\frac{m_{z}}{\sqrt{m_{x}^{2}+m_{y}^{2}+m_{z}^{2}}}\right).$$

On the one hand, when ${\hat{\theta }}_{0}=0$, it is obvious that the vector ***m*** is parallel to the *z* axis, which means it is meaningless to estimate azimuth, so we stop estimating. On the other hand, when ${\hat{\theta }}_{0}\neq 0$, the vector ***m*** is not parallel to the *z* axis, hence

(13) $$m_{x}^{2}+m_{y}^{2}\neq 0.$$

Similarly, according to the relationship between the projection of vector ***m*** onto the *xy* plane and the *x* axis in Figure 2, it can be known that

(14) $$\cos\phi_{0}=\frac{m_{x}}{\sqrt{m_{x}^{2}+m_{y}^{2}}}.$$

Furthermore, according to the definition of azimuth, the estimated value of azimuth is

(15) $${\hat{\phi }}_{0}=\{\begin{array}{c} \arccos\left(\frac{m_{x}}{\sqrt{m_{x}^{2}+m_{y}^{2}}}\right)\begin{array}{ccc} & & m_{y}\geq 0 \end{array}\\ -\arccos\left(\frac{m_{x}}{\sqrt{m_{x}^{2}+m_{y}^{2}}}\right)\begin{array}{ccc} & & m_{y}\leq 0 \end{array} \end{array}.$$

### 3.4. Actual Angle Estimation Algorithm

**Figure 3:**
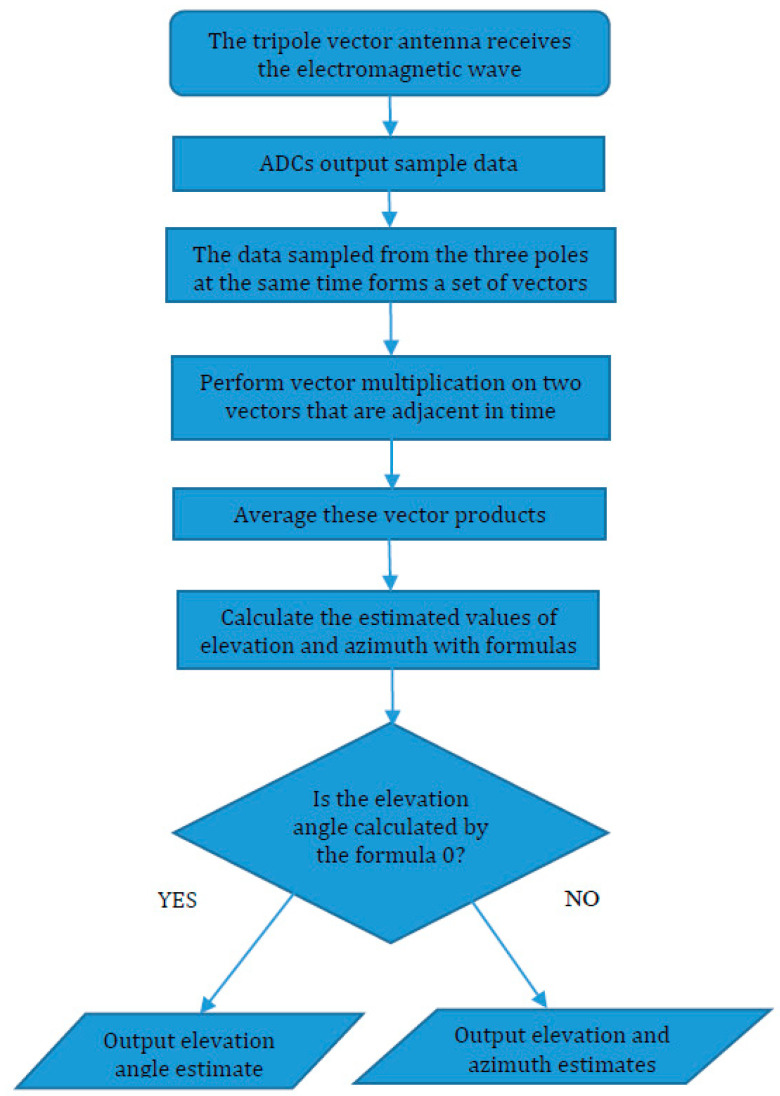
A flowchart about obtaining the direction of arrival (DOA).

The implementation of the algorithm is shown in Figure 3. In order to decrease the effect of noise, we can take the average value with multiple estimates. Obviously, the calculation of the anti-trigonometric function in Equations (12) and (15) are the most complex calculation steps in the whole estimation algorithm, and these steps are also the most sensitive to noise. Therefore, when calculating the vector ***m***, taking the average value with *K* sample snapshots is an effective action to reduce the influence of noise, because this operation is before the calculation of anti-trigonometric functions. Then, the estimated value of ***m*** is

(16) $$\bar{m}=\frac{1}{K}\sum_{k=1}^{K}m\left(k\right)={\left[\begin{array}{ccc} {\bar{m}}_{x} & {\bar{m}}_{y} & {\bar{m}}_{z} \end{array}\right]}^{T}.$$

Correspondingly, DOA estimates are modified to

(17) $${\hat{\theta }}_{0}=\arccos\left(\frac{{\bar{m}}_{z}}{\sqrt{{\bar{m}}_{x}^{2}+{\bar{m}}_{y}^{2}+{\bar{m}}_{z}^{2}}}\right)$$

(18) $${\hat{\phi }}_{0}=\{\begin{array}{c} \arccos\left(\frac{{\bar{m}}_{x}}{\sqrt{{\bar{m}}_{x}^{2}+{\bar{m}}_{y}^{2}}}\right)\begin{array}{ccc} & & {\bar{m}}_{y}\geq 0 \end{array}\\ -\arccos\left(\frac{{\bar{m}}_{x}}{\sqrt{{\bar{m}}_{x}^{2}+{\bar{m}}_{y}^{2}}}\right)\begin{array}{ccc} & & {\bar{m}}_{y}\leq 0 \end{array} \end{array}.$$

## 4. Performance Analysis

### 4.1. Probability Distribution of Estimated Angles

Since there is noise in the snapshot, the estimated angles ${\hat{\theta }}_{0}$ and ${\hat{\phi }}_{0}$ are considered as random variables. In order to find out the Probability Density Function (PDF), the PDFs of *m_x_*, *m_y_*, and *m_z_* need to be calculated first.

Considering noise components in received signals, we plug Equations (1)–(3) into Equations (8)–(10) respectively, and obtain *m_x_*, *m_y_*, and *m_z_* with noise. Since the three variables have the same structure, only the PDF of *m_x_* is analyzed. For *m_x_*, which can be expanded as

(19) $$m_{x}=m_{x0}+P_{x}+Q_{x}$$

where

(20) $$m_{x0}=y\left(2k\right)z\left(2k+1\right)-z\left(2k\right)y\left(2k+1\right)$$

(21) $$P_{x}=n_{y}\left(2k\right)n_{z}\left(2k+1\right)-n_{z}\left(2k\right)n_{y}\left(2k+1\right)$$

(22) $$\begin{array}{ll} Q_{x} & =n_{y}\left(2k\right)E_{z}^{}\left(2k+1\right)+n_{z}\left(2k+1\right)E_{y}^{}\left(2k\right)\\ & -n_{z}\left(2k\right)E_{y}^{}\left(2k+1\right)-n_{y}\left(2k+1\right)E_{z}^{}\left(2k\right) \end{array}.$$

Obviously, *m_x0_* is ideal value without noise, but *P_x_* and *Q_x_* have noise. Apparently, noise snapshot *n_x_*, *n_y_*, and *n_z_* are Gaussian random variables with a mean 0 and variance $\sigma_{x}^{2}$, $\sigma_{y}^{2}$, and $\sigma_{z}^{2}$ respectively.

Since *Q_x_* is the sum of multiple independent Gaussian random variables, *Q_x_* is also a random variable of Gaussian distribution. According to Equation (22), it is not difficult to find out the mean and variance are respectively:

(23) $$E\left[Q_{x}\right]=0$$

(24) $$\begin{array}{ll} D\left[Q_{x}\right]= & \sigma_{Q_{x}}^{2}=\sigma_{y}^{2}E_{z}^{2}\left(2k+1\right)+\sigma_{z}^{2}E_{y}^{2}\left(2k\right)\\ & \begin{array}{ccc} & & +\sigma_{z}^{2}E_{y}^{2}\left(2k+1\right)+\sigma_{y}^{2}E_{z}^{2}\left(2k\right) \end{array} \end{array}.$$

Therefore, the PDF of *Q_x_* is:

(25) $$f_{Q_{x}}\left(Q_{x}\right)=\frac{1}{\sqrt{2\pi }\sigma_{Q_{x}}^{}}\exp\left(-\frac{Q_{x}^{2}}{2\sigma_{Q_{x}}^{2}}\right).$$

In order to obtain the PDF of *P_x_*, two related theorems are given in advance.

**Theorem 1.**  *If two independent random variables x and y obey the Gaussian distribution with mean 0 and variance*$\sigma_{x}^{2}$*,*$\sigma_{y}^{2}$*the PDF of the variable z = xy is*:

(26) $$f_{z}\left(z\right)=g\left(z\right)=\frac{K_{0}\left(\frac{\left|z\right|}{\sigma_{x}^{}\sigma_{y}^{}}\right)}{\pi \sigma_{x}^{}\sigma_{y}^{}}$$

*where the function K_0_ is the modified Bessel function of the second kind of order 0.*

**Theorem 2**  **[22].***If the PDFs of two independent random variables x and y are f_x_(x) and f_y_(y) respectively, the PDF of the variable z = x + y is*:

(27) $$f_{z}\left(z\right)=\int_{-\infty }^{+\infty }f_{x}\left(t\right)f_{y}\left(z-t\right)dt.$$

Looking back to Equation (21), function *P_x_* is the difference of two independent components, and each independent component is the product of two independent Gaussian variables. Hence, using Theorems 1 and 2, we can get the PDF of the variable *P_x_*:

(28) $$f_{P_{x}}\left(P_{x}\right)=\int_{-\infty }^{+\infty }g\left(t\right)g\left(t-P_{x}\right)dt=\frac{1}{2\sigma_{z}\sigma_{y}}\exp\left(\frac{-\left|P_{x}\right|}{\sigma_{z}\sigma_{y}}\right).$$

According to Equation (19), and using Theorem 2 again, we can get the PDF of variable *m_x_*; similarly, we can get PDFs of *m_y_*, *m_z_*.

(29) $$\begin{array}{l} f_{m_{x}}\left(m_{x}\right)=\int_{-\infty }^{+\infty }f_{P_{x}}\left(t\right)f_{Q_{x}}\left(m_{x}-m_{x0}-t\right)dt\\ =\int_{-\infty }^{+\infty }\frac{1}{2\sqrt{2\pi }\sigma_{Q_{x}}\sigma_{z}\sigma_{y}}\exp\left(-\frac{{\left(m_{x}-t-m_{x0}\right)}^{2}}{2\sigma_{Q_{x}}^{2}}-\frac{\left|t\right|}{\sigma_{z}\sigma_{y}}\right)dt \end{array}$$

(30) $$\begin{array}{l} f_{m_{y}}\left(m_{y}\right)=\int_{-\infty }^{+\infty }f_{P_{y}}\left(t\right)f_{Q_{y}}\left(m_{y}-m_{y0}-t\right)dt\\ =\int_{-\infty }^{+\infty }\frac{1}{2\sqrt{2\pi }\sigma_{Q_{y}}\sigma_{z}\sigma_{x}}\exp\left(-\frac{{\left(m_{y}-t-m_{y0}\right)}^{2}}{2\sigma_{Q_{y}}^{2}}-\frac{\left|t\right|}{\sigma_{z}\sigma_{x}}\right)dt \end{array}$$

(31) $$\begin{array}{l} f_{m_{z}}\left(m_{z}\right)=\int_{-\infty }^{+\infty }f_{P_{z}}\left(t\right)f_{Q_{z}}\left(m_{z}-m_{z0}-t\right)dt\\ =\int_{-\infty }^{+\infty }\frac{1}{2\sqrt{2\pi }\sigma_{Q_{z}}\sigma_{x}\sigma_{y}}\exp\left(-\frac{{\left(m_{z}-t-m_{z0}\right)}^{2}}{2\sigma_{Q_{z}}^{2}}-\frac{\left|t\right|}{\sigma_{x}\sigma_{y}}\right)dt \end{array}$$

According to Equation (24), similarly, we can define $\sigma_{Q_{y}}^{2}$, $\sigma_{Q_{z}}^{2}$ as follows

(32) $$\sigma_{Q_{y}}^{2}=\sigma_{x}^{2}E_{z}^{2}\left(2k+1\right)+\sigma_{z}^{2}E_{x}^{2}\left(2k\right)+\sigma_{z}^{2}E_{x}^{2}\left(2k+1\right)+\sigma_{x}^{2}E_{z}^{2}\left(2k\right)$$

(33) $$\sigma_{Q_{z}}^{2}=\sigma_{x}^{2}E_{y}^{2}\left(2k+1\right)+\sigma_{y}^{2}E_{x}^{2}\left(2k\right)+\sigma_{y}^{2}E_{x}^{2}\left(2k+1\right)+\sigma_{x}^{2}E_{y}^{2}\left(2k\right).$$

In order to simplify Equations (29)–(31), we replace exp(−|t|) with a function, which fits well with the original function. This alternative function is

(34) $$h\left(t\right)=a_{1}e^{\frac{-t^{2}}{b_{1}}}+a_{2}e^{\frac{-t^{2}}{b_{2}}}$$

where *a*_1_ = 0.4652, *b*_1_ = 0.5524, *a*_2_ = 0.4396, *b*_2_ = 1.911, and max |exp(−|t|) − *h*(*t*)| is less than 0.03, which means these two functions fit well.

In this way, Equations (29)–(31) can be replaced as:

(35) $$\begin{array}{ll} f_{m_{x}}\left(m_{x}\right) & =\frac{a_{1}}{4\sigma_{Q_{x}}^{2}\sigma_{z}^{2}\sigma_{y}^{2}}\sqrt{\frac{\sigma_{z}^{2}\sigma_{y}^{2}+2b_{1}\sigma_{Q_{x}}^{2}}{b_{1}}}\exp\left(-\frac{2b_{1}\sigma_{Q_{x}}^{2}\sigma_{z}^{2}\sigma_{y}^{2}}{\sigma_{z}^{2}\sigma_{y}^{2}+2b_{1}\sigma_{Q_{x}}^{2}}{\left(m_{x}-m_{x0}\right)}^{2}\right)\\ & +\frac{a_{2}}{4\sigma_{Q_{x}}^{2}\sigma_{z}^{2}\sigma_{y}^{2}}\sqrt{\frac{\sigma_{z}^{2}\sigma_{y}^{2}+2b_{2}\sigma_{Q_{x}}^{2}}{b_{2}}}\exp\left(-\frac{2b_{2}\sigma_{Q_{x}}^{2}\sigma_{z}^{2}\sigma_{y}^{2}}{\sigma_{z}^{2}\sigma_{y}^{2}+2b_{2}\sigma_{Q_{x}}^{2}}{\left(m_{x}-m_{x0}\right)}^{2}\right) \end{array}$$

(36) $$\begin{array}{ll} f_{m_{y}}\left(m_{y}\right) & =\frac{a_{1}}{4\sigma_{Q_{y}}^{2}\sigma_{z}^{2}\sigma_{x}^{2}}\sqrt{\frac{\sigma_{z}^{2}\sigma_{x}^{2}+2b_{1}\sigma_{Q_{y}}^{2}}{b_{1}}}\exp\left(-\frac{2b_{1}\sigma_{Q_{y}}^{2}\sigma_{z}^{2}\sigma_{x}^{2}}{\sigma_{z}^{2}\sigma_{x}^{2}+2b_{1}\sigma_{Q_{y}}^{2}}{\left(m_{y}-m_{y0}\right)}^{2}\right)\\ & +\frac{a_{2}}{4\sigma_{Q_{y}}^{2}\sigma_{z}^{2}\sigma_{x}^{2}}\sqrt{\frac{\sigma_{z}^{2}\sigma_{x}^{2}+2b_{2}\sigma_{Q_{y}}^{2}}{b_{2}}}\exp\left(-\frac{2b_{2}\sigma_{Q_{y}}^{2}\sigma_{z}^{2}\sigma_{x}^{2}}{\sigma_{z}^{2}\sigma_{x}^{2}+2b_{2}\sigma_{Q_{y}}^{2}}{\left(m_{y}-m_{y0}\right)}^{2}\right) \end{array}$$

(37) $$\begin{array}{ll} f_{m_{z}}\left(m_{z}\right) & =\frac{a_{1}}{4\sigma_{Q_{z}}^{2}\sigma_{x}^{2}\sigma_{y}^{2}}\sqrt{\frac{\sigma_{x}^{2}\sigma_{y}^{2}+2b_{1}\sigma_{Q_{z}}^{2}}{b_{1}}}\exp\left(-\frac{2b_{1}\sigma_{Q_{z}}^{2}\sigma_{x}^{2}\sigma_{y}^{2}}{\sigma_{x}^{2}\sigma_{y}^{2}+2b_{1}\sigma_{Q_{z}}^{2}}{\left(m_{z}-m_{z0}\right)}^{2}\right)\\ & +\frac{a_{2}}{4\sigma_{Q_{z}}^{2}\sigma_{x}^{2}\sigma_{y}^{2}}\sqrt{\frac{\sigma_{x}^{2}\sigma_{y}^{2}+2b_{2}\sigma_{Q_{z}}^{2}}{b_{2}}}\exp\left(-\frac{2b_{2}\sigma_{Q_{z}}^{2}\sigma_{x}^{2}\sigma_{y}^{2}}{\sigma_{x}^{2}\sigma_{y}^{2}+2b_{2}\sigma_{Q_{z}}^{2}}{\left(m_{z}-m_{z0}\right)}^{2}\right) \end{array}.$$

In order to get PDFs of ${\hat{\theta }}_{0}$ and ${\hat{\phi }}_{0}$, we quote two more related theorems.

**Theorem 3**  **[22].** *If* u=g(x,y,z) *, and* z(u,x,y) *is the inverse function of u on z, u has continuous partial derivative to x, y, z, the joint PDF of x, y, z is* f(x,y,z) *, hence the PDF of u is*

(38) $$g\left(u\right)=\int_{-\infty }^{+\infty }\int_{-\infty }^{+\infty }f\left(z\left(u,x,y\right),x,y\right)\left|\frac{\partial z}{\partial u}\right|dxdy.$$

**Theorem 4**  **[22].** *If* u=g(x,y) *, and* y(u,x) *is the inverse function of u on y, u has continuous partial derivative to x, y, the joint PDF of x, y is* f(x,y) *, hence the PDF of u is*

(39) $$g\left(u\right)=\int_{-\infty }^{+\infty }f\left(y\left(u,x\right),x\right)\left|\frac{\partial y}{\partial u}\right|dx.$$

If we define the joint PDF of random variables (*m_x_*, *m_y_*, *m_z_*) is *f*_1_(*m_x_*, *m_y_*, *m_z_*), then

(40) $$f_{1}\left(x,y,z\right)=f_{m_{x}}\left(x\right)f_{m_{y}}\left(y\right)f_{m_{z}}\left(z\right).$$

Replacing *m_x_*, *m_y_*, and *m_z_* with *x*, *y*, and *z*, according to Equation (12) and Theorem 3, the PDF of the estimated elevation angle ${\hat{\theta }}_{0}$ can be obtained as

(41) $$f_{\theta }\left({\hat{\theta }}_{0}\right)=\int_{-\infty }^{+\infty }\int_{-\infty }^{+\infty }f_{1}\left(x,y,z\left(x,y,{\hat{\theta }}_{0}\right)\right)\left|\frac{\partial \left(x,y,z\right)}{\partial \left({\hat{\theta }}_{0},x,y\right)}\right|dxdy$$

where

(42) $$\left|\frac{\partial z}{\partial {\hat{\theta }}_{0}}\right|=\frac{\sqrt{x^{2}+y^{2}}}{\sin^{2}{\hat{\theta }}_{0}}.$$

So,

(43) $$f_{\theta }\left({\hat{\theta }}_{0}\right)=\int_{-\infty }^{+\infty }\int_{-\infty }^{+\infty }f_{m_{x}}\left(x\right)f_{m_{y}}\left(y\right)f_{m_{z}}\left(\sqrt{x_{}^{2}+y_{}^{2}}\cot{\hat{\theta }}_{0}\right)\frac{\sqrt{x^{2}+y^{2}}}{\sin^{2}{\hat{\theta }}_{0}}dxdy.$$

In the same way, if the joint PDF of random variables (*m_x_*, *m_y_*) is

(44) $$f_{2}\left(x,y\right)=f_{m_{x}}\left(x\right)f_{m_{y}}\left(y\right),$$

according to Equation (15) and Theorem 4, the PDF of the estimated azimuth ${\hat{\phi }}_{0}$ can be obtained as

(45) $$f_{\phi }\left({\hat{\phi }}_{0}\right)=\int_{-\infty }^{+\infty }f_{2}\left(x,y\left(x,{\hat{\phi }}_{0}\right)\right)\left|\frac{\partial y}{\partial {\hat{\phi }}_{0}}\right|dx$$

where

(46) $$\left|\frac{\partial y}{\partial {\hat{\phi }}_{0}}\right|=\left|x\right|\sec^{2}{\hat{\phi }}_{0}.$$

Hence

(47) $$f_{\phi }\left({\hat{\phi }}_{0}\right)=\int_{-\infty }^{+\infty }f_{m_{x}}\left(x\right)f_{m_{y}}\left(x\tan{\hat{\phi }}_{0}\right)\left|x\right|\sec^{2}{\hat{\phi }}_{0}dx$$

after simplification,

(48) $$f_{\phi }\left({\hat{\phi }}_{0}\right)=\sec^{2}{\hat{\phi }}_{0}\sum_{i=1}^{2}\sum_{j=1}^{2}q_{ij}\left({\hat{\phi }}_{0}\right)\left(2-\Phi \left(\frac{-\mu_{ij}}{\sigma_{ij}}\right)-\Phi \left(\frac{\mu_{ij}}{\sigma_{ij}}\right)\right)$$

where $\Phi \left(x\right)=\int_{-\infty }^{x}\frac{\exp\left(-t^{2}\right)}{\sqrt{2\pi }}dt$ and

(49) $$q_{ij}\left({\hat{\phi }}_{0}\right)=r_{ij}\exp\left(s_{ij}{\left(m_{y0}^{}-\tan^{}{\hat{\phi }}_{0}m_{x0}^{}\right)}^{2}\right)\{\begin{array}{c} i=1,2\\ j=1,2 \end{array}$$

(50) $$r_{ij}=\frac{a_{i}a_{j}}{8\pi \sigma_{Q_{x}}\sigma_{Q_{y}}\sigma_{z}^{2}\sigma_{y}\sigma_{x}}\sqrt{\pi \left(\frac{2b_{i}\sigma_{Q_{x}}^{2}\sigma_{z}\sigma_{y}}{\sigma_{z}\sigma_{y}+2b_{i}\sigma_{Q_{x}}^{2}}+\frac{2b_{j}\sigma_{Q_{y}}^{2}\sigma_{z}\sigma_{x}}{\sigma_{z}\sigma_{x}+2b_{j}\sigma_{Q_{y}}^{2}}\tan^{2}{\hat{\phi }}_{0}\right)}$$

(51) $$\mu_{ij}=\frac{b_{i}\sigma_{Q_{x}}^{2}\sigma_{y}\left(\sigma_{z}\sigma_{x}+2b_{j}\sigma_{Q_{y}}^{2}\right)m_{x0}+b_{j}\sigma_{Q_{y}}^{2}\sigma_{x}\left(\sigma_{z}\sigma_{y}+2b_{i}\sigma_{Q_{x}}^{2}\right)m_{y0}\tan{\hat{\phi }}_{0}}{b_{i}\sigma_{Q_{x}}^{2}\sigma_{y}\left(\sigma_{z}\sigma_{x}+2b_{j}\sigma_{Q_{y}}^{2}\right)+b_{j}\sigma_{Q_{y}}^{2}\sigma_{x}\left(\sigma_{z}\sigma_{y}+2b_{i}\sigma_{Q_{x}}^{2}\right)\tan^{2}{\hat{\phi }}_{0}}$$

(52) $$\sigma_{ij}=\frac{1}{2}\sqrt{\frac{\left(\sigma_{z}\sigma_{y}+2b_{i}\sigma_{Q_{x}}^{2}\right)\left(\sigma_{z}\sigma_{x}+2b_{j}\sigma_{Q_{y}}^{2}\right)}{b_{i}\sigma_{Q_{x}}^{2}\sigma_{y}\sigma_{z}\left(\sigma_{z}\sigma_{x}+2b_{j}\sigma_{Q_{y}}^{2}\right)+b_{j}\sigma_{Q_{y}}^{2}\sigma_{x}\sigma_{z}\left(\sigma_{z}\sigma_{y}+2b_{i}\sigma_{Q_{x}}^{2}\right)\tan^{2}{\hat{\phi }}_{0}}}$$

(53) $$s_{ij}=\frac{2b_{i}b_{j}\sigma_{Q_{x}}^{2}\sigma_{Q_{y}}^{2}\sigma_{z}\sigma_{y}\sigma_{x}}{b_{i}\sigma_{Q_{x}}^{2}\sigma_{y}\left(\sigma_{z}\sigma_{x}+2b_{j}\sigma_{Q_{y}}^{2}\right)+b_{j}\sigma_{Q_{y}}^{2}\sigma_{x}\left(\sigma_{z}\sigma_{y}+2b_{i}\sigma_{Q_{x}}^{2}\right)\tan^{2}{\hat{\phi }}_{0}}.$$

Clearly, Φ(x) is a cumulative distribution function of the standard normal distribution with an integral. For the sake of simplicity, we use function I(x) to replace Φ(x) if max|Φ(x) − I(*x*)| is less than 0.01,

(54) $$I\left(x\right)=\frac{1}{1+\exp\left(-1.701x\right)}.$$

Hence,

(55) $$f_{\phi }\left({\hat{\phi }}_{0}\right)=\sec^{2}{\hat{\phi }}_{0}\sum_{i=1}^{2}\sum_{j=1}^{2}q_{ij}\left({\hat{\phi }}_{0}\right)\left(2-I\left(\frac{-\mu_{ij}}{\sigma_{ij}}\right)-I\left(\frac{\mu_{ij}}{\sigma_{ij}}\right)\right).$$

### 4.2. Statistical Characteristics of Angle Estimatior

The statistical characteristics of angle estimators are the main indicators to measure the estimation performance. In order to analyze the statistical characteristics of angle estimates, we first study the distribution characteristics of the vector $\bar{m}$, which is obtained by Equation (16). According to Section 4.1, the estimated $\bar{m}$ is symmetrically distributed in space about the accurate value ***m***_0_; as a result, the mean value of $\bar{m}$ should be equal to the accurate value ***m***_0_, namely

(56) $$E\left[{\bar{m}}_{x}\right]=m_{x0}$$

(57) $$E\left[{\bar{m}}_{y}\right]=m_{y0}$$

(58) $$E\left[{\bar{m}}_{z}\right]=m_{z0}.$$

Meanwhile, let $\sigma_{mx}^{2}$, $\sigma_{my}^{2}$ and $\sigma_{mz}^{2}$ as the variance of *m_x_*, *m_y_*, and *m_z_* and ${\bar{\sigma }}_{mx}^{2}$, ${\bar{\sigma }}_{my}^{2}$ and ${\bar{\sigma }}_{mz}^{2}$ as the variance of ${\bar{m}}_{x}$, ${\bar{m}}_{y}$ and ${\bar{m}}_{z}$ according to the relationship among variables above, we can obtain

(59) $${\bar{\sigma }}_{mx}^{2}=\frac{\sigma_{mx}^{2}}{K}$$

(60) $${\bar{\sigma }}_{my}^{2}=\frac{\sigma_{my}^{2}}{K}$$

(61) $${\bar{\sigma }}_{mz}^{2}=\frac{\sigma_{mz}^{2}}{K}.$$

Obviously, when the number of snapshots *K* tends to infinity, ${\bar{\sigma }}_{mx}^{2}$, ${\bar{\sigma }}_{my}^{2}$ and ${\bar{\sigma }}_{mz}^{2}$ are all close to 0.

According to above conclusion, using Chebyshev’s theorem, the following inequalities are established:

(62) $$P\left\{\left|{\bar{m}}_{x}-m_{x0}\right|\geq \varepsilon_{1}\right\}\leq \frac{{\bar{\sigma }}_{mx}^{2}}{\varepsilon_{1}^{2}}$$

(63) $$P\left\{\left|{\bar{m}}_{y}-m_{y0}\right|\geq \varepsilon_{2}\right\}\leq \frac{{\bar{\sigma }}_{my}^{2}}{\varepsilon_{2}^{2}}$$

(64) $$P\left\{\left|{\bar{m}}_{z}-m_{z0}\right|\geq \varepsilon_{3}\right\}\leq \frac{{\bar{\sigma }}_{mz}^{2}}{\varepsilon_{3}^{2}}$$

where *ε*_1_, *ε*_2_, and *ε*_3_ are arbitrary positive numbers. 

Clearly, the probability for ${\bar{m}}_{x}$, ${\bar{m}}_{y}$ and ${\bar{m}}_{z}$ deviate from *m_x_*_0_, *m_y_*_0_, and *m_z_*_0_ tends to 0 respectively if the number of snapshots *K* is very large. Combining Equations (16) and (17), we can get

(65) $$\underset{K\to \infty }{\lim}\arccos\left(\frac{{\bar{m}}_{z}}{\sqrt{{\bar{m}}_{x}^{2}+{\bar{m}}_{y}^{2}+{\bar{m}}_{z}^{2}}}\right)=\arccos\left(\frac{m_{z0}}{\sqrt{m_{x0}^{2}+m_{y0}^{2}+m_{z0}^{2}}}\right)=\theta_{0}$$

(66) $$\{\begin{array}{c} \underset{K\to \infty }{\lim}\arccos\left(\frac{{\bar{m}}_{x}}{\sqrt{{\bar{m}}_{x}^{2}+{\bar{m}}_{y}^{2}}}\right)=\arccos\left(\frac{m_{x0}}{\sqrt{m_{x0}^{2}+m_{y0}^{2}}}\right)=\phi_{0}\left({\bar{m}}_{y}\geq 0\right)\\ \underset{K\to \infty }{\lim}-\arccos\left(\frac{{\bar{m}}_{x}}{\sqrt{{\bar{m}}_{x}^{2}+{\bar{m}}_{y}^{2}}}\right)=\arccos\left(\frac{m_{x0}}{\sqrt{m_{x0}^{2}+m_{y0}^{2}}}\right)=\phi_{0}\left({\bar{m}}_{y}\leq 0\right) \end{array}.$$

Therefore, we can conclude that our algorithm is an asymptotic unbiased estimation, and the estimated value will become closer to the true value as the number of snapshots increases.

### 4.3. Analysis of Algorithm Complexity

MUSIC is a DOA estimation method based on subspace, which uses eigenvalue decomposition to the covariance matrix and searches peaks by spatial spectrum. Its advantage is that *D* different signals can be estimated at the same time. However, the disadvantage is that the *N*-element array must be used and results in heavy computation load, where *N* > *D*.

The computation load of the interferometer algorithm is relatively small, but the amount of operation increases significantly with the number of array elements. In addition, if the correction for amplitude and phase errors is considered, the amount of operation would further increase.

Compared with above these two algorithms, the proposed algorithm based on a tripole vector antenna has a significant advantage in computation. When *K* snapshots are used for estimation, the new algorithm here only needs to complete multiplication in 6*K* − 1 times, addition in 6*K* − 1 times, division in 2 times, square root in 2 times, and anti-cosine function in 2 times. 

In details, the square root operation is calculated by Newton iterative method, and the iterative formula is

(67) $$x_{n+1}=\frac{b}{2x_{n}}+\frac{x_{n}}{2}\begin{array}{cc} & x_{0}=b \end{array}$$

where *b* is a radicand. Since *A* means iteration times and *x_A_* is the square root of *b*, there are *A* times of addition, *A* times of multiplication, and *A* times of division in one square root operation. On the other hand, we calculate the inverse cosine function by Taylor’s expansion

(68) $$\arccos x=\frac{\pi }{2}-\sum_{n=0}^{M}\left(\frac{\left(2n\right)!}{2^{{}_{2n}}{\left(n!\right)}^{2}}\right)\frac{x^{2n+1}}{2n+1}=\frac{\pi }{2}-\sum_{n=0}^{M}a_{n}x^{2n+1}\left(\left|x\right|\leq 1\right).$$

So, the amount of an anti-cosine function computation consists of (*M* + 1) (*M* + 2) times of multiplication, n+1 times of addition. 

In conclusion, the computation load of the proposed algorithm includes *M*^2^ + 3*M* + 1 + 2*A* + 6*K* times of multiplication, *n* + 2*A* + 6*K* times of addition, 2 + 2*A* times of division. The computation load of each algorithm can be seen in Table 1.

Where *Q* is spectral peak search times of azimuth and *I* is that of elevation.

It can be seen from Table 1 that compared with existing methods, our algorithm of angle estimation based on a single tripole vector antenna has obvious advantages in computational complexity.

## 5. Simulations

In this section, we present some numerical results to assess the performance of our algorithm. For a comparison, we investigate the performance of MUSIC [11], Interferometer [9], and our proposed algorithm.

In the following simulations, we consider an L-shaped array consisting of three parallel dipoles used for the interferometer, and three triple vector antennas used for both MUSIC and the proposed algorithm. The spectral peak search interval of the MUSIC algorithm is 0.1 degree. In addition, we assume that the DOA of the incident EM wave is $\theta_{0}=\varphi_{0}=30^{\circ }$, and the frequency of the EM wave is 1 GHz.

For a statistical comparison, we define the root mean square error (RMSE) as

(69) $$\mathrm{RMSE}=\sqrt{\frac{1}{J}\sum_{j=1}^{J}\left[{\left(\phi_{0}-{\hat{\phi }}_{0j}\right)}^{2}+{\left(\theta_{0}-{\hat{\theta }}_{0j}\right)}^{2}\right]}$$

where $\phi_{0}$ and $\theta_{0}$ are the actual values of azimuth and elevation angle, respectively. In addition, ${\hat{\phi }}_{0j}$ and ${\hat{\theta }}_{0j}$ are the estimated values of azimuth and elevation angle in the *j*-th Monte Carlo trial where *j* = 1, 2, ..., *J*.

We also consider the statistical characteristics of the estimation error for the proposed algorithm. The estimation error is defined as the difference between the estimated value and true value, that is

(70) $$\left\{ \begin{array}{l} \Delta \theta =\theta_{0}-{\hat{\theta }}_{0}\\ \Delta \phi =\phi_{0}-{\hat{\phi }}_{0} \end{array}\right..$$

Their mean and variance are

(71) $$\left\{ \begin{array}{l} E\left[\Delta \theta \right]=\frac{1}{J}\sum_{j=1}^{J}\left(\theta_{0}-{\hat{\theta }}_{0j}\right)\\ D\left[\Delta \theta \right]=E^{2}\left[\Delta \theta \right]-E{\left[\Delta \theta^{2}\right]}^{} \end{array}\right.$$

(72) $$\left\{ \begin{array}{l} E\left[\Delta \phi \right]=\frac{1}{J}\sum_{j=1}^{J}\left(\phi_{0}-{\hat{\phi }}_{0j}\right)\\ D\left[\Delta \phi \right]=E^{2}\left[\Delta \phi \right]-E{\left[\Delta \phi^{2}\right]}^{} \end{array}\right..$$

In addition, the signal-to-noise ratio is defined as

(73) $$\mathrm{SNR}\;=\;10\mathrm{lg}\frac{P_{s}}{P_{n}}$$

where *P_s_* and *P_n_* represent the effective power of signal and noise, respectively.

### 5.1. Simulation 1: Statistical Characteristics of Angle Error

**Figure 4:**
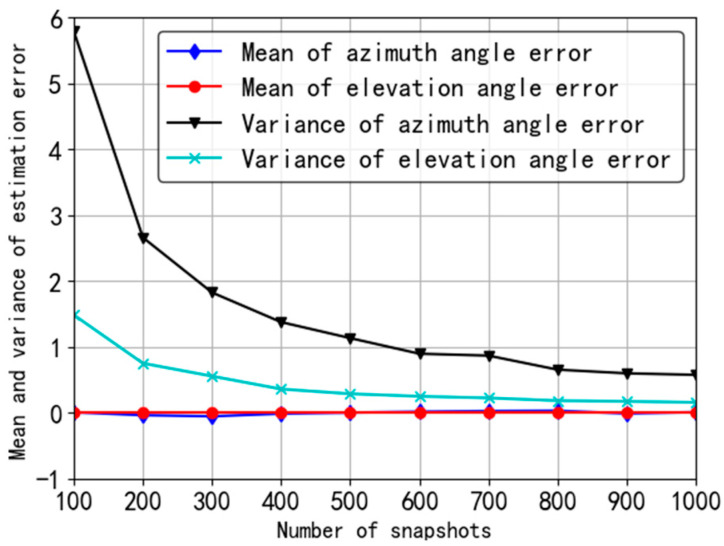
Simulation of angle estimation error.

In the first simulation, we mainly introduce the statistical characteristics of the estimation error of the proposed algorithm according to Equations (71) and (72). Supposing *J* = 200 and SNR = 10 dB, the results are shown in Figure 4.

In Figure 4, the mean of the estimation error is almost always 0 even in the case of small number of snapshots, which illustrates that the estimation method can be considered unbiased and the error mean is not sensitive to the number of snapshots. However, the variance of estimation error is relatively large when the number of snapshots is small. Increasing the number of snapshots can reduce the variance. Furthermore, the variance of azimuth is always larger than that of elevation angle.

### 5.2. Simulation 2: Comparison of RMSE Performance Versus Different SNR

**Figure 5:**
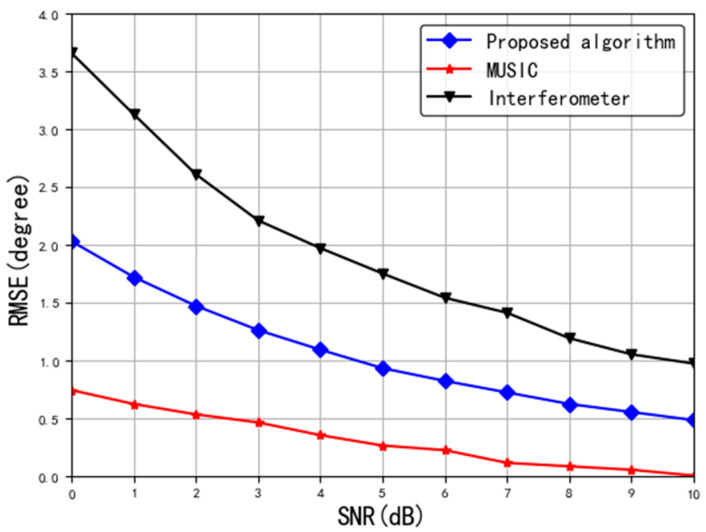
Relationship between estimation error and SNR.

The second simulation illustrates the comparison of the performance among three kinds of DOA estimation algorithms, which are phase interferometer, MUSIC, and the proposed tripole vector antenna method. In the simulation, *K* = 1000, *J* = 200, and SNR varies from 0 to 10 dB. The results are shown in Figure 5.

It depicts in Figure 5 that the MUSIC algorithm has the best performance followed by the proposed algorithm here. In addition, due to the polarization mismatch between the EM wave and the antenna for interferometer, the input SNR loss is 3 dB and leads to an estimation error almost twice that of the proposed method.

### 5.3. Simulation 3: Comparison of RMSE Performance Versus Different Snapshots

**Figure 6:**
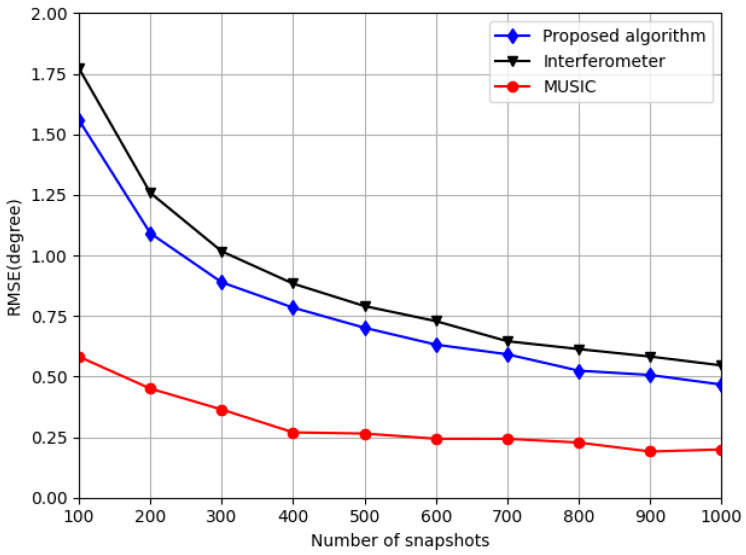
Relationship between estimation error and the number of snapshots.

The third simulation compares the performance of the three algorithms under different snapshots. In this simulation, SNR = 10 dB, *J* = 200, and *K* varies from 100 to 1000. The results are shown in Figure 6.

As illustrated in Figure 6, when the number of snapshots is the same, the performance of MUSIC is the best, followed by the proposed algorithm and the interferometer. This result is very similar to the simulation result in Section 5.2, which shows that increasing number of snapshots is equal to improving the SNR for the proposed algorithm.

### 5.4. Simulation 4: RMSE for Different DOAs in Degrees

In order to fully demonstrate the estimated performance of the proposed algorithm at various angles, we simulated all angles under the condition of 10 dB and 1000 snapshots, and the results are as follows.

**Figure 7:**
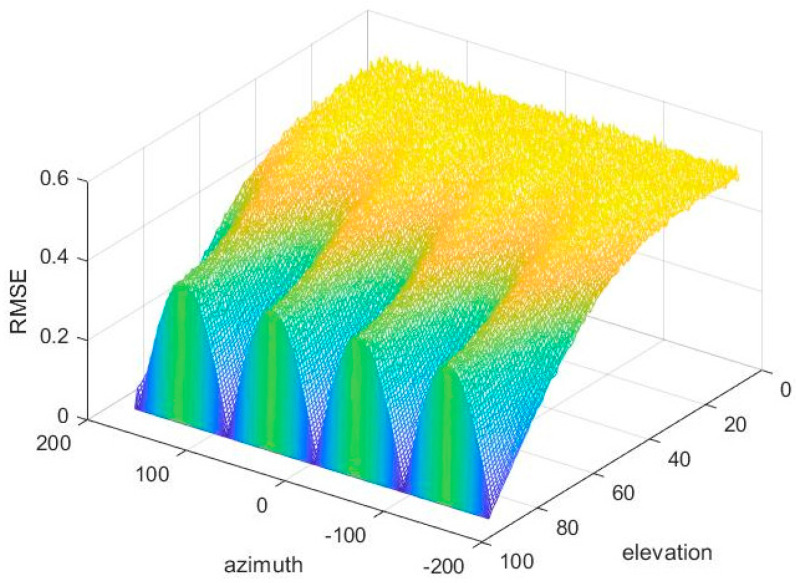
Root mean square error (RMSE) for different DOAs in degrees.

According to Figure 7, estimate errors are different in various incident angles. In the situation of invariant azimuth, as a decline of elevation, RMSE decreases; when the elevation stays invariant, the RMSE varies periodically with the change of azimuth, the RMSE is relatively small when azimuth is kπ/2, and it is relatively large when kπ/2 + π/4. The above simulation results are demonstrated from Equations (17) and (18). According to Equation (17), the elevation angle depends on $\frac{{\bar{m}}_{x}^{2}+{\bar{m}}_{y}^{2}}{{\bar{m}}_{z}^{2}}$; according to Equation (18), azimuth depends on $\frac{{\bar{m}}_{x}^{2}}{{\bar{m}}_{x}^{2}+{\bar{m}}_{y}^{2}}$, which has nothing to do with ${\bar{m}}_{z}$.

### 5.5. Simulation 5: Algorithm Complexity

**Figure 8:**
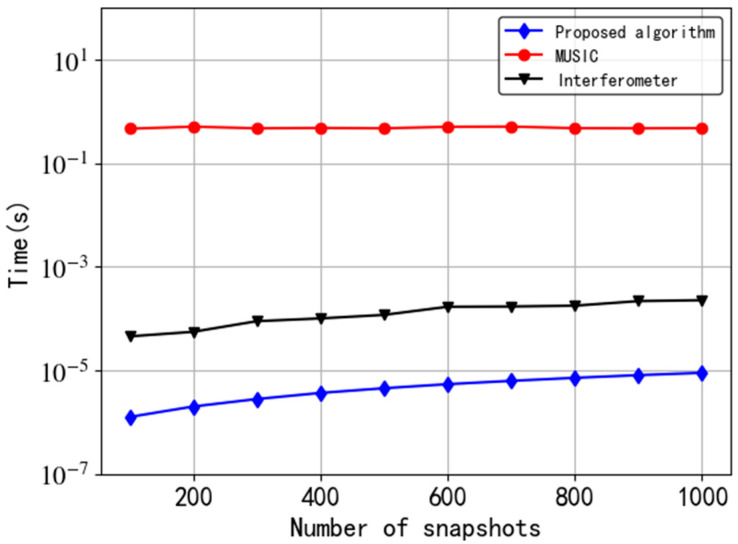
Relationship between operation time and the number of snapshots.

In this simulation, we record the running time of different algorithms in MATLAB, which is obtained by taking the average of the running time of multiple Monte Carlo experiments. The version of MATLAB is 2018b. The CPU of the computer running the experiment is i7-7700HQ with a clock frequency of 2.8 GHz. The simulation results are shown in Figure 8.

Figure 8 presents that the speed of the proposed algorithm and the interferometer algorithm are both much faster than that of the MUSIC algorithm. In detail, the running time of our algorithm is at least 5 orders of magnitude lower than that of MUSIC algorithm. Furthermore, as the number of snapshots increases, the calculation time of the interferometer algorithm is approximately 40 to 200 times that of the proposed algorithm. Obviously, the significant advantage of the proposed tripole antenna algorithm is the low computational complexity.

## 6. Conclusions

We investigate the DOA estimation with a tripole vector antenna for a single nonlinearly polarized EM wave. Unlike traditional dipole antennas, tripole vector antennas can completely capture the electric field of EM waves, which makes it possible to estimate the DOA directly through the geometric relationship between the signal propagation direction and the tripole vector antenna. In the proposed method, the antenna array is not required so that hardware cost is reduced. In addition, many complex operations are not required, such as spectrum search and matrix eigenvalue decomposition, which means the proposed algorithm has extremely fast operation speed with satisfactory accuracy. In the future, we will modify the new algorithm to enlarge the suitable application fields. In addition, combining the proposed algorithm with other schemes to obtain better performance in DOA estimation will also be considered.

## Figures and Tables

**Table 1:** The computational complexity of the algorithm. MUSIC: Multiple Signal Classification.

Algorithm	Times of Multiplication	Times of Addition	Times of Division
MUSIC [11]	(2*N* − 1) (*N* − *D*) *IQ* + *NK*	(2*N* − 1) (*N* − *D*) *IQ* + (2*N* − 1) *K*	1
Interferometer [9]	8 + *A* + 4 (*M* + 1) (*M* + 2) + 2*K* + 0.5 (*N* − 2) *K*log_2_*K*	*A* + 1 + 2*K* + 4 (*n* + 1) + *K*log_2_*K*	6 + *A*
Proposed Algorithm	*M*^2^ + 3*M* + 1 + 2*A* + 6*K*	*n* + 2*A* + 6*K*	2 + 2*A*
